# Secondary Plasma Cell Leukemia: A Case Report

**DOI:** 10.7759/cureus.8693

**Published:** 2020-06-19

**Authors:** Neeraja Swaminathan, Gabor Varadi

**Affiliations:** 1 Internal Medicine, Albert Einstein Medical Center, Philadelphia, USA; 2 Hematology and Oncology, Albert Einstein Medical Center, Philadelphia, USA

**Keywords:** plasma cell leukemia, multiple myeloma

## Abstract

Plasma cell leukemia (PCL) is a rare and aggressive variant of myeloma and has a poor prognosis. It needs prompt recognition in order to institute timely treatment. Given its relatively low incidence, it is an evolving area of research as well. This case report describes a patient with PCL in the setting of a previously treated myeloma. The report also reviews the clinicopathologic, cytogenetic, and immunophenotypic characteristics of PCL and its management.

## Introduction

Plasma cell leukemia (PCL) is an unusual and aggressive form of myeloma that can occur de novo or as a secondary transformation of multiple myeloma (MM). PCL incidence accounts for around 2-4% of plasma cell malignancies of which primary PCL (pPCL) constitutes the majority of cases (60-70%). However, with an increase in the survival rate of myeloma patients, secondary PCL (sPCL) is becoming more frequent [1,2]. As with myeloma, PCL is also more common in African Americans [3]. pPCL tends to present at a younger age compared to sPCL. pPCL and sPCL are distinct entities, but both have a poor prognosis. The overall median survival is 6-11 months for sPCL occurring in the setting of relapsed or refractory myeloma having worse outcomes. Male-to-female distribution in both pPCL and secondary sPCL is around 3:2 [1]. Clinical presentation of PCL includes anemia, thrombocytopenia, renal dysfunction, hypercalcemia, bone pain, lytic lesions, infections, and hepatosplenomegaly, etc. PCL has distinct pathological features that can distinguish it from myeloma. Given the rarity of this disease, diagnostic and treatment criteria are still being investigated.

## Case presentation

**Figure 1:**
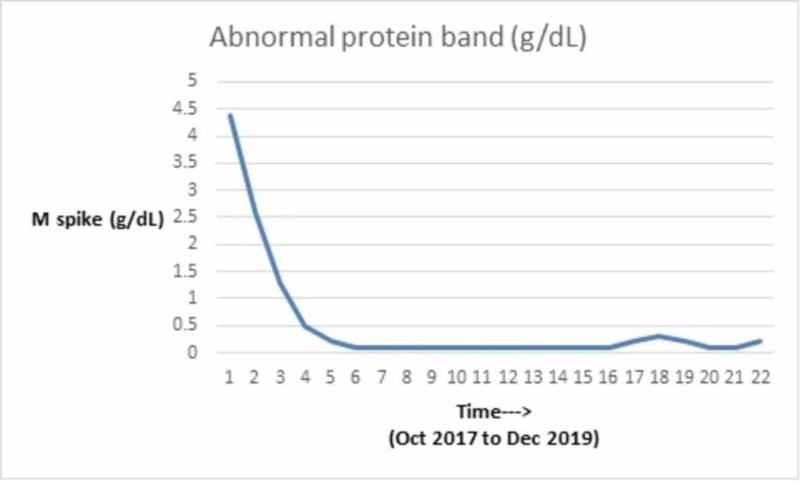
Graphical trend of M-spike M-spike: monoclonal spike

**Figure 2:**
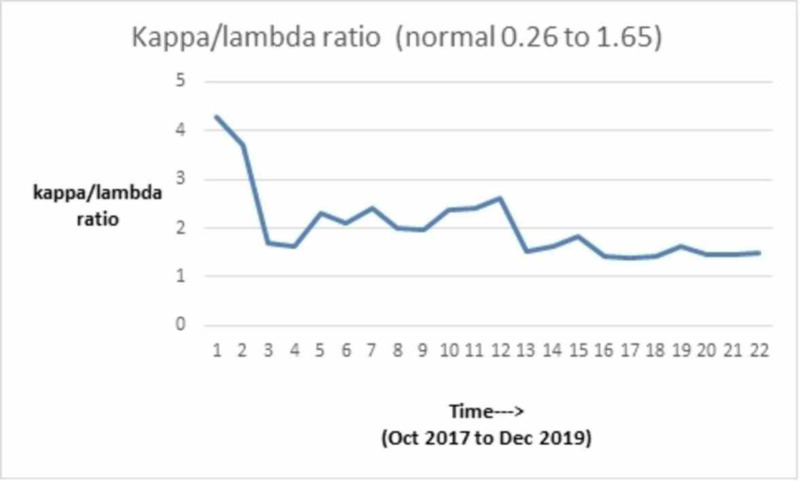
Graphical trend of kappa/lambda ratio

**Figure 3:**
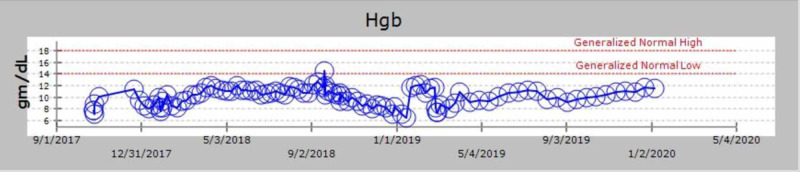
Graphical trend of hemoglobin Hgb: hemoglobin

**Figure 4:**
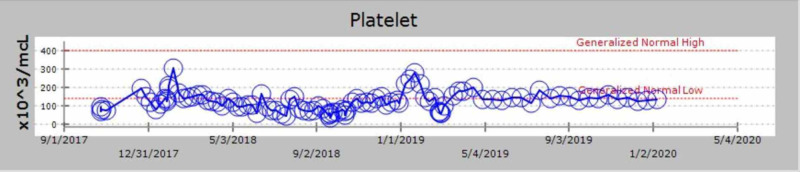
Graphical trend of platelet count

**Figure 5:**
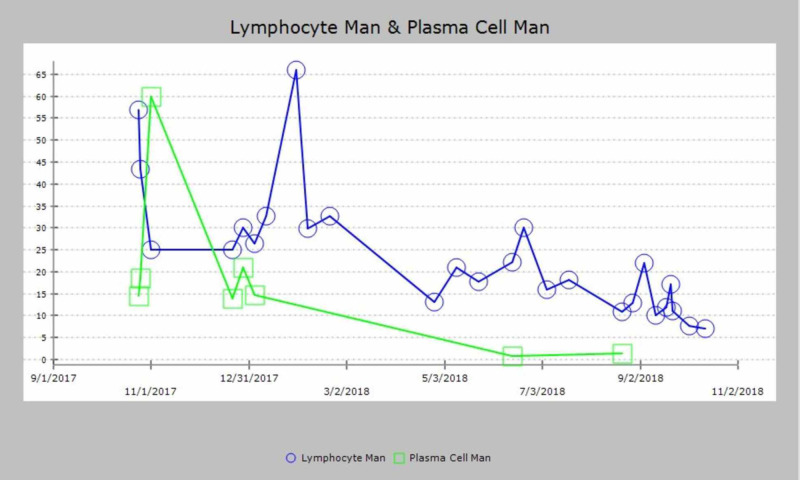
Graphical trend of lymphocyte and plasma cell count

**Figure 6:**
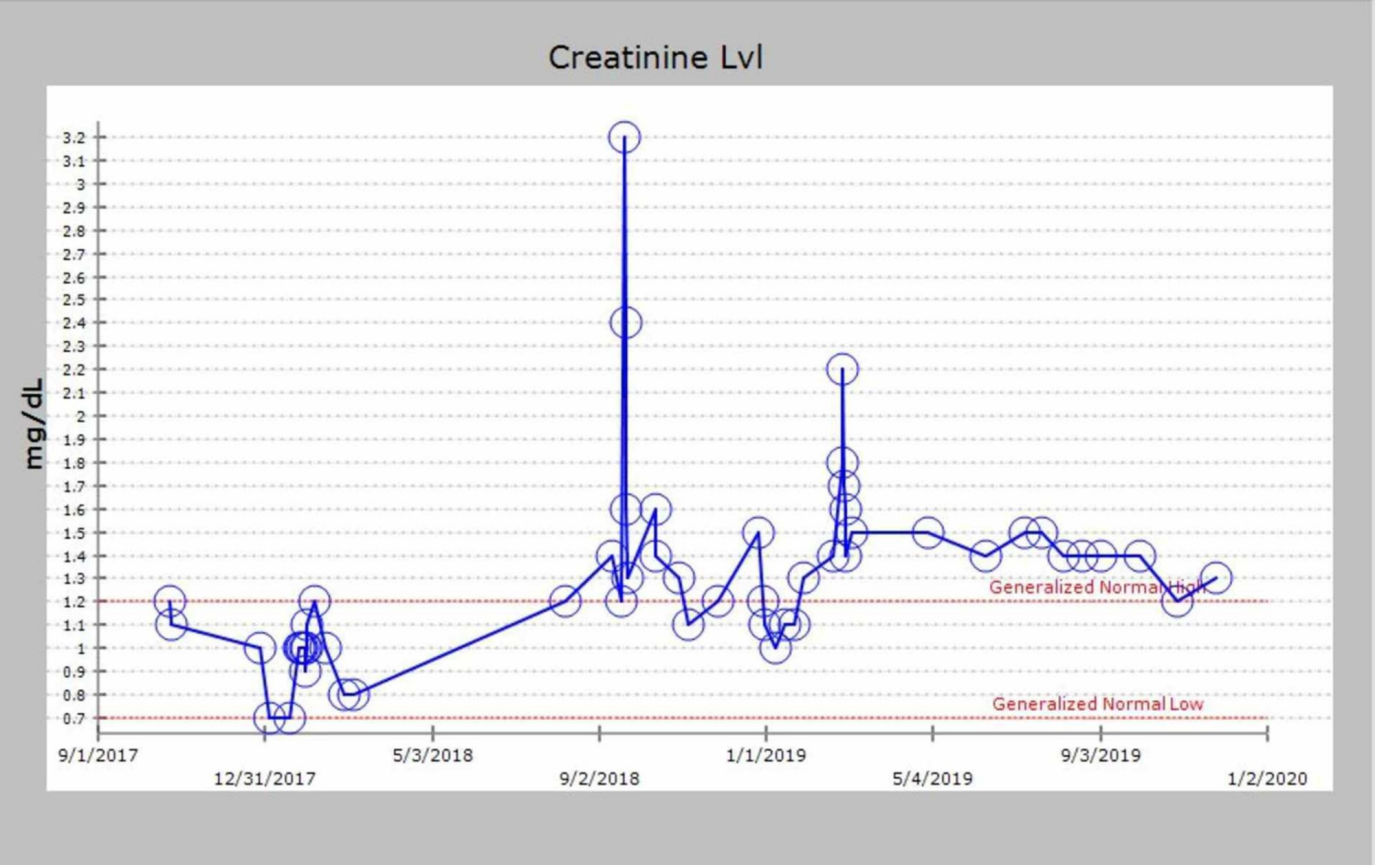
Graphical trend of creatinine

We discuss the case of a 71-year-old African-American man with a past medical history of MM diagnosed in 2000. He received induction chemotherapy with four cycles of adriamycin, vincristine, and dexamethasone and subsequently underwent an autologous stem cell transplant in 2001. He also had a history of substance use (tobacco and ethanol), alcohol-related gastritis, chronic systolic heart failure with recovered ejection fraction. In October 2017, he presented with complaints of lower back pain. On examination, he did not have any signs of spinal cord compression. He had mild pallor but no lymphadenopathy or hepatosplenomegaly. Given his myeloma history, there was a concern for relapse. He underwent imaging as part of the workup, which revealed multiple chronic lytic lesions and multilevel vertebral body compression fractures. Investigations were notable for anemia (hemoglobin of 7.1 g/L) and thrombocytopenia (platelet count of 78 x 10^3^/microliter). His peripheral smear showed leukocytosis with predominant lymphocytes and plasmacytosis (WBC count of 16.6 x 10^3^/microliter with greater than 20% plasma cells). Other notable lab studies included serum creatinine of 1.1 mg/dL, calcium 9 mg/dL, lactate dehydrogenase (LDH) 113 IU/L, beta-2 microglobulin 16.40 mg/L. Serum protein electrophoresis revealed immunoglobulin G (IgG) kappa with 4.4 g M-spike (monoclonal protein), and urinalysis was positive for Bence Jones protein. Bone marrow biopsy showed diffuse involvement with more than 65% plasma cells with flow cytometry positive for cluster of differentiation (CD) 138, kappa, and lambda. Table 1 shows the trend of serum electrophoresis results; the graphical trends of the abnormal protein band (M-spike) are seen in Figure 1, Kappa/Lambda ratio in Figure 2, blood counts in Figures 3, 4, 5 and creatinine in Figure 6 respectively.

**Table 1:** Serum electrophoresis results IgG: immunoglobulin G; IgA: immunoglobulin A; IgM: immunoglobulin M

	Alpha 1 (g/dL)	Alpha 2 (g/dL)	Gamma (g/dL)	Abnormal protein band (g/dL)	Albumin/globulin ratio (normal range: 1-2.5)	Kappa/lambda ratio (normal range: 0.26-1.65)	Beta-2 microglobulin (mg/L)	IgG (mg/dL)	IgA (mg/dL)	IgM (mg/dL)
17-Oct	0.5	1.4	3.9	4.4	0.5	4.3	16.5	5637	26	14
18-Jan	0.4	0.9	2.7	2.6	0.6	3.71		3304	11	11
18-Feb	0.3	0.9	1.5	1.3	1.4	1.71		1749	12	13
18-Apr	0.3	0.9	0.8	0.5	1.6	1.61	2.2	942	14	18
18-May	0.3	0.8	0.4	0.2	2.1	2.31		455		
18-Jun	0.3	0.9	0.3	0.1	1.9	2.11		360	11	10
18-Jul	0.2	0.6	0.3	0.1	2.3	2.4		304	8	6
18-Aug	0.2	0.7	0.3	0.1	2.3	2		269	7	6
18-Sep	0.3	0.7	0.3	0.1	2.5	1.96		292	8	7
18-Oct	0.4	1	0.2	0.1	1.8	2.39		245	8	7
18-Nov	0.3	0.8	0.2	0.1	1.8	2.4				
18-Dec	0.4	0.9	0.2	0.1	2.2	2.63		225	9	6
19-Feb	0.4	0.9	0.4	0.1	1.7	1.52		380	28	7
19-Mar	0.4	0.9	0.3	0.1	1.5	1.61		375	30	8
19-Apr	0.4	0.6	0.6	0.1	1.6	1.82				
19-May	0.4	0.7	0.6	0.1	1.4	1.43				
19-Jun	0.5	0.7	0.7	0.2	1.2	1.39		762	45	11
19-Jul	0.5	0.6	0.8	0.3	1.2	1.41		925	67	21
19-Sep	0.4	0.7	0.7	0.2	1.5	1.62				
19-Oct	0.4	0.8	0.8	0.1	1.5	1.44		919	64	14
19-Nov	0.4	0.7	0.9	0.1	1.4	1.45		1120	72	17
19-Dec	0.4	0.9	1	0.2	1.3	1.48				

Induction chemotherapy was initiated with lenalidomide (oral 25 mg on days 1-14 of the 21-day cycle), weekly bortezomib (1.3 mg/m^2^ subcutaneous), and dexamethasone (40 mg oral weekly). He was also given supportive therapy with hydration, allopurinol (300 mg oral daily), antimicrobial prophylaxis (acyclovir 400 mg oral twice daily for herpes prophylaxis), bisphosphonates (zoledronic acid 4 mg intravenous once a month) and denosumab (120 mg subcutaneous once a month).

Within a week of initiating therapy, he was admitted to a local hospital with pneumonia. He was critically ill with pneumonia and septic shock. He also had acute onset worsening of cardiac function. There was a concern for cardiotoxicity of bortezomib; however, his cardiac function returned to baseline with the resolution of his septic shock. He returned to the office in December 2017 after recovering from his critical illness.

At this time, he was still recovering from acute kidney injury and hence was switched to cyclophosphamide (450 mg oral on days 1, 8, 15, and 22), bortezomib (2.25 mg subcutaneous), and dexamethasone (40 mg oral on days 1, 8, 15, and 22) (CyBorD) regimen. After the initiation of this regimen, he started showing good clinical response. In July 2018, after six cycles of therapy, he showed no evidence of leukemia/plasma cell neoplasm although a mild elevation in his paraprotein level remained. This amounted to a very good partial response.

CyBrorD was continued from December 2017 to March 2019. The option of a second stem cell transplant was also considered during his course and he was referred for the same. He was deemed not to be a re-transplant candidate in view of abnormal pulmonary function tests likely due to chronic smoking and concurrent substance use disorders. Hence, he was started on maintenance bortezomib in April 2019, which he still receives every other week and has been tolerating well. He continues to have a trace band in the gamma region on electrophoresis with mild elevation of the serum-free light chain ratio. However, he has not shown any sign of progressive myeloma or relapsing PCL.

Despite the aggressive nature of the disease and poor outcomes usually associated with it, our case highlights the example of a patient who has responded well to treatment and is in remission now.

## Discussion

Plasma cell malignancies include the following entities: classic MM, extramedullary plasmacytoma without MM, solitary plasmacytoma of the bone, and PCL. The incidence of PCL in patients with MM is 2-4% and it accounts for 0.3% of leukemias. Extramedullary involvement is more common in pPCL as compared to PCL secondary to MM [4-7].

PCL is defined as the presence of greater than 2 x 109/liter plasma cells or >20% plasmacytosis on a differential WBC count. It is a rare and aggressive disease and can either be a primary malignant plasma cell proliferation or a secondary leukemic transformation of previously diagnosed MM [8]. MM, pPCL, and sPCL are biologically distinct entities. PCL occurs as a result of increased/decreased expression of factors that promote the growth of tumor cells outside the bone marrow microenvironment. sPCL is characterized by the multistep accumulation of genetic changes in a patient with advanced relapsed or refractory myeloma whereas pPCL has these changes de novo. Immunophenotypic expression is similar in PCL and MM for CD 38, CD 138, and CD 2, CD 3, CD 16, CD 10, CD 13, and CD 15. PCLs, however, are more likely to express CD 20, CD23, CD 44, CD 45 and not to express CD 56, CD 9, CD 71, CD 117, human leukocyte antigen (HLA) DR, neural cell adhesion molecule (NCAM), and leukocyte function-associated antigen 1 (LFA-1), which helps differentiate it from MM. pPCL and sPCL share similar immunophenotype except for CD 28, which is expressed with greater frequency in sPCL. CD 28 is a poor prognostic factor in MM and is associated with plasma cell proliferation, disease progression, and chemotherapy resistance [6,7,9,10]. PCL cells are more often likely to be non-hyperploid compared to MM. Chromosomal translocations involving immunoglobulin heavy chain (IgH), 17p deletion, 1p21 deletion, and 1q21 amplifications are seen in both pPCL and sPCL. pPCL is typically positive for t(11;14); although this can also be seen in sPCL, it is more frequently associated with t(4;14) and t(14;16) [11]. All of these features summarized in Table 2.

**Table 2:** Immunophenotypic characteristics of MM, pPCL, and sPCL MM: multiple myeloma; pPCL: primary plasma cell leukemia; sPCL: secondary plasma cell leukemia; IgH: immunoglobulin heavy chain; HLA: human leukocyte antigen; NCAM: neural cell adhesion molecule; LFA-1: leukocyte function-associated antigen 1

Features of MM, pPCL, and sPCL	Features of pPCL AND sPCL	Features of pPCL	Features of sPCL
Presence of CD38, CD 138 CD 2, CD 3, CD 10, CD 13 CD 15, CD 16	Presence of CD 20, CD 23, CD 44, CD 45, IgH translocations, MYC translocations/amplifications, 17 p deletion, 1p21 deletion, 1q21 amplification; lack of CD 9, CD 56, CD 71, CD 117, HLA DR, NCAM, LFA-1	Presence of t(11;14)	Presence of CD 28, t(4;14), t(14;16)

As in our patient, sPCL occurs due to the dissemination of tumor cells into the peripheral bloodstream from the bone marrow causing the resultant leukemic transformation. It occurs due to change in expression of adhesion molecules and chemokine receptors and due to several molecular aberrations as noted above. This leads to inhibition of apoptosis, immune surveillance evasion of the tumor cells, and extramedullary extension and proliferation [12,13].

Like other leukemias, PCL can also present with overt clinical signs such as hepatosplenomegaly and lymphadenopathy, but this is infrequent and occurs in only up to 15% of the patients [2]. The presence of plasma cells can be confirmed with flow cytometry positivity for markers as noted above, especially CD 38 and CD 138. Valuable information can be obtained from chromosomal and cytogenetic analysis because it correlates with patient outcomes. Favorable/intermediate prognosis is conferred by hyperdiploid karyotype, normal karyotype, t(11;14) (q32;q32). Unfavorable factors include hypodiploid karyotype, complex karyotype, del(13q14), del(17p13), del(1p21), amp 1q21, and MYC translocations/amplifications [12].

sPCL is frequently resistant to many agents since it often can be an end-stage manifestation of a myeloma patient who has received several lines of treatment. Since PCL is a rare entity, there is no consensus regarding its treatment. Combination chemotherapy especially with proteasome inhibitors and immunomodulatory drugs followed by stem cell transplantation is the standard treatment currently.

The newer treatment options include proteasome inhibitors and immunomodulators in addition to stem cell transplantation. The use of proteasome inhibitors such as bortezomib has been studied in pPCL, and they have shown a treatment response of 79%, two-year progression-free survival of 55%, and a two-year overall survival of 40%. Similarly, with immunomodulators, lenalidomide has been studied in pPCL patients, and in combination with dexamethasone, it has been shown to have similar results. Recommendations for treatment approach in PCL are to initiate induction chemotherapy with a triplet regimen containing a novel agent such as VRd (bortezomib, lenalidomide, dexamethasone) or KRd (carfilzomib, lenalidomide, dexamethasone). In patients with extensive disease burden, regimens such as VDT-PACE, which comprises bortezomib, dexamethasone, thalidomide, cisplatin, doxorubicin, cyclophosphamide, and etoposide, can be used. However, in elderly and frail patients, milder regimens such as CyBorD (cyclophosphamide, bortezomib, dexamethasone) or PAD (bortezomib, doxorubicin, dexamethasone) are preferred. Consolidation with autologous stem cell transplantation (ASCT) is recommended post-induction therapy. Allogenic stem cell transplants have been shown to be inferior in comparison with ASCT; they have a higher mortality rate and should be done in clinical trial settings. If patients are not transplant-eligible, they should be treated with combination therapy with indefinite maintenance in order to control the disease.

Future scope includes studying other newer drugs as a potential therapeutic alternative, such as elotuzumab, daratumumab, chimeric antigen receptor (CAR), T cell therapy, and venetoclax. As with many oncological conditions, treatment should be tailored based on the patient’s profile. Patients with PCL should be considered for newer treatment options, and trials and collaboration are important in developing newer treatment modalities [12,14].

## Conclusions

PCL is an aggressive variant of plasma dyscrasia and requires prompt initiation of treatment. This case report highlights the ways to recognize this condition and also reviews its management. Given the condition's rarity, it is infrequently reported in the literature and there are limited guidance and recommendations on treatment. Hence, it is definitely a subject that merits further research and reporting.
